# lncRNAs and circRNAs: Emerging Players in Pediatric Medulloblastoma Pathology

**DOI:** 10.2174/0109298673354413250325073924

**Published:** 2025-04-15

**Authors:** Ozal Beylerli, Elmar Musaev, Tatiana Ilyasova, Albert Sufianov

**Affiliations:** 1 Central Research laboratory, Bashkir State Medical University, 3 Lenin Street, Ufa, 450008, Russia;; 2 Department of Oncology, Sechenov First Moscow State Medical University, Moscow, Russia;; 3 Department of Internal Diseases, Bashkir State Medical University, 3 Lenin Street, Ufa, 450008, Russia;; 4 Educational and Scientific Institute of Neurosurgery, Рeoples’ Friendship University of Russia (RUDN University), Moscow, Russia;; 5 Department of Neurosurgery, Sechenov First Moscow State Medical University (Sechenov University), Moscow, Russia

**Keywords:** lncRNAs, circRNAs, pediatric brain tumors, medulloblastomas, pathogenesis, cell proliferation

## Abstract

Medulloblastomas (MBs) are the most common malignant brain tumors in children, marked by aggressive growth, molecular heterogeneity, and a high propensity for cerebrospinal dissemination. Despite advancements in conventional treatments - surgery, chemotherapy, and radiation therapy—substantial challenges persist, including debilitating long-term toxicities and emerging resistance to therapy. This review examines the multifaceted roles of non-coding RNAs (ncRNAs) - particularly long non- coding RNAs (lncRNAs) and circular RNAs (circRNAs) - in pediatric medulloblastoma pathogenesis, diagnosis, and therapeutic targeting. NcRNAs exert robust regulatory effects on gene expression by modulating signaling pathways, acting as miRNA sponges, and controlling the expression of oncogenic or tumor-suppressive genes. In this study, we focus on notable examples of lncRNAs (*e.g.*, HOTAIR, TP73-AS1) and circRNAs (*e.g.*, circ-SKA3, circ_63706) implicated in fundamental oncogenic processes, such as cell proliferation, apoptosis, metastasis, and stem cell maintenance. We also discuss their subgroup-specific roles, emphasizing high-risk groups, such as Sonic Hedgehog (SHH) and Group 3 medulloblastomas. In parallel, we explore the potential of ncRNAs to serve as diagnostic/prognostic biomarkers, given their tissue-specific expression, stability, and detectability in biological fluids like the Cerebrospinal Fluid (CSF). Finally, we review emerging therapeutic strategies, including antisense oligonucleotides, RNA sponges, and CRISPR-based editing, aimed at disrupting oncogenic ncRNA functions or reinforcing tumor-suppressive pathways. While these strategies hold promise, major hurdles include functional redundancy, optimizing *in vivo* delivery, and mitigating off-target effects. By detailing these challenges and outlining future research directions, this review underscores the revolutionary potential of ncRNA-focused diagnostics and therapies for managing pediatric medulloblastomas, offering new paths for improving survival outcomes and quality of life in affected children.

## 1 INTRODUCTION

Medulloblastomas (MBs) account for approximately 20% of all pediatric brain tumors, originating from embryonal cells in the cerebellum [1-3]. Although modern neurosurgical techniques, combined with radiation and high-dose chemotherapy, have improved survival rates, the aggressiveness of MB and its propensity for metastasis continue to pose significant clinical challenges. Moreover, the life-altering toxicities associated with intensive regimens-ranging from neurocognitive deficits to significant endocrine and psychosocial complications-necessitate the development of targeted, less harmful therapeutic strategies.

## 2 MOLECULAR SUBGROUPS OF MEDULLOBLASTOMA

MBs exhibit notable molecular and clinical heterogeneity, leading to their subdivision into four major subgroups: Wingless (WNT), Sonic Hedgehog (SHH), Group 3, and Group 4 [4]. Characterized by an active WNT signaling pathway, they are usually manifested in children older than three years and demonstrate relatively favorable outcomes, with 5-year survival rates exceeding 95% [5-7]. New research distinguishes WNT-α and WNT-β subtypes, further refining patient stratification [8]. Driven by constitutive activation of the SHH pathway, these tumors predominantly appear in infants and adults and exhibit intermediate prognosis (around 75% 5-year survival). Gene expression and methylation data have delineated four SHH variants - SHH-α, SHH-β, SHH-γ, and SHH-δ - highlighting the intragroup complexity of SHH MB. Often associated with MYC amplification, Group 3 tumors represent roughly 25% of MBs and have the worst overall prognosis. They frequently present with metastatic disease. Although the most common subgroup, Group 4, remains less understood. These tumors display a variety of genetic and epigenetic alterations and present diverse clinical outcomes [9-11]. Understanding these subgroups on a molecular level not only refines diagnosis and prognostication but also supports the development of targeted treatments that could alleviate the toxic burden on pediatric patients.

## 3 NCRNAS IN MEDULLOBLASTOMA RESEARCH

**Fig. (1):**
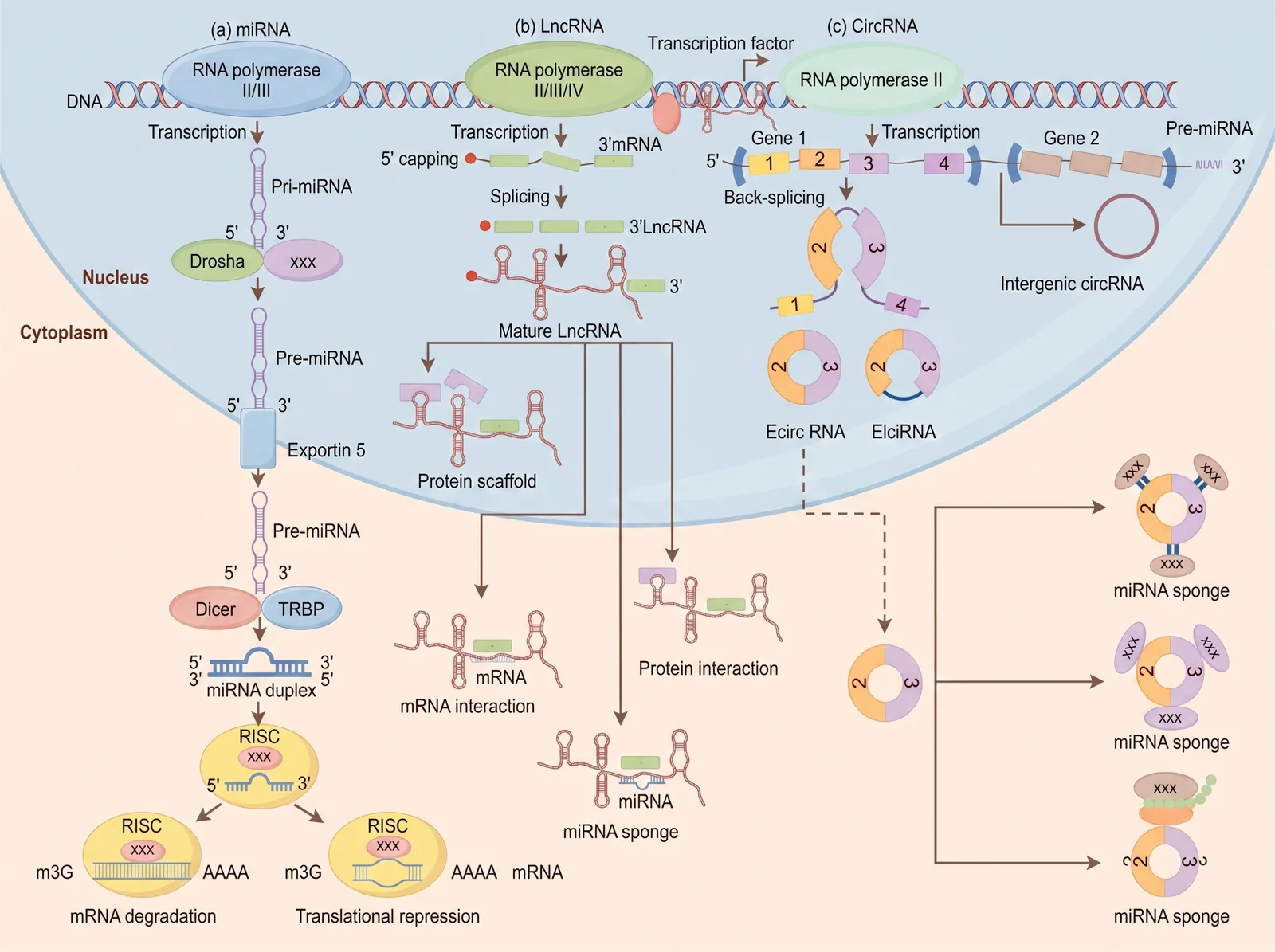
Biogenesis of ncRNAs. NcRNAs, which include miRNAs, lncRNAs, and circRNAs, are generated through distinct processing pathways. While miRNAs are derived from hairpin-structured precursors, lncRNAs are generally transcribed from intergenic or intronic regions, and circRNAs form closed-loop structures through back-splicing events.

In pursuit of more precise diagnostic and therapeutic options, attention has turned toward ncRNAs, including miRNAs, lncRNAs, and circRNAs. These ncRNAs significantly influence gene expression (Fig. **1**) [12-15]. By regulating processes, such as cell proliferation, invasion, and stemness, ncRNAs appear integral to MB biology. Typically, longer than 200 nucleotides, these molecules modulate transcriptional and post-transcriptional mechanisms, often forming complexes with proteins or acting as sponges for miRNAs. Defined by a covalently closed loop structure, circRNAs are resistant to exonuclease-mediated degradation and can serve as robust miRNA sponges, regulators of transcription, or modulators of protein function. Recent studies underscore the subgroup specificity of many ncRNAs, suggesting roles both as biomarkers for risk stratification and as therapeutic targets. This review consolidates existing literature to provide an in-depth survey of how lncRNAs and circRNAs contribute to MB pathogenesis, the techniques used to discover and characterize them, and how these findings could be translated into clinical applications.

These lncRNAs and circRNAs exert a profound influence over a myriad of cellular processes, such as cell proliferation, differentiation, apoptosis, and metastasis, each fundamental to both the progression of cancer and potential therapeutic targeting. The increasing recognition of the roles of lncRNAs and circRNAs offers a novel lens through which the biology of pediatric medulloblastomas can be understood and manipulated for clinical benefit. This review article aims to thoroughly examine how lncRNAs and circRNAs contribute to the oncogenesis, progression, and treatment resistance of pediatric medulloblastomas. We will delve into recent discoveries regarding the various types of lncRNAs and circRNAs, exploring their mechanisms of action and their dynamic interactions within the cellular and molecular landscapes of medulloblastoma [16, 17]. Furthermore, the potential of lncRNAs and circRNAs as diagnostic and prognostic biomarkers will be evaluated, highlighting their capacity to refine the accuracy of diagnoses and predict therapeutic outcomes more reliably.

Additionally, the review will discuss the emerging therapeutic implications of targeting lncRNAs and circRNAs in medulloblastoma treatment. By altering lncRNA and circRNA profiles, new therapeutic avenues may open that can potentially shift current treatment paradigms and lead to more personalized and effective interventions. By exploring these aspects, this review seeks to underline the transformative potential of lncRNA and circRNA research in revolutionizing the management and outcomes of pediatric medulloblastoma. It aims to set a direction for future research and clinical applications, emphasizing the necessity of continued exploration in this vital area of pediatric oncology, where innovative research could lead to significant advancements in treatment and patient care.

### 3.1 lncRNAs in Medulloblastoma

Studies consistently demonstrate aberrant expression of lncRNAs in MB (Table **1**). These lncRNAs can modulate oncogenic or tumor-suppressive signaling through RNA-RNA, RNA-protein, and RNA-DNA interactions.

### 3.2 Major Oncogenic LncRNAs and Mechanisms

Research by Shi *et al.* highlighted SPRY4-IT1 as an oncogenic driver in MB [18]. When siRNA was used to silence SPRY4-IT1 in Daoy MB cells, real- time quantitative PCR (qPCR) confirmed a marked reduction in its expression. Proliferation assays (CCK-8 and colony formation) demonstrated a decrease in cell growth, while matrigel invasion assays revealed significantly impaired metastatic potential. Western blot analysis showed downregulation of MMP-2 and MMP-9, two enzymes essential for Extracellular Matrix (ECM) breakdown. Thus, SPRY4-IT1 appears to facilitate MB aggressiveness by modifying ECM remodeling processes. Given its role in promoting invasion, SPRY4-IT1 is a potential target for therapies aiming to curb metastasis. Functional validation of SPRY4-IT1 in additional MB cell lines and Patient-Derived Xenograft (PDX) models could solidify its candidacy for clinical translation. LOXL1-AS1 is significantly increased in MB tissues, correlating with more advanced disease [19]. Moreover, knockdown in D283 and D341 cell lines curtailed cell growth, colony formation, and invasiveness. Flow cytometry revealed an S-phase arrest upon LOXL1-AS1 inhibition, suggestive of compromised cell cycle regulation. LOXL1-AS1 modulates phosphorylation events in the PI3K/AKT pathway, a key oncogenic signaling cascade driving cell survival and EMT. Xenograft experiments confirmed that suppressing LOXL1-AS1 leads to smaller, less invasive tumors, reinforcing its status as a promising therapeutic target. Laneve *et al.* identified linc-NeD125 as a ceRNA in Group 4 MB [20]. By sequestering miR-19a-3p, miR-19b-3p, and miR-106a-5p, linc-NeD125 derepresses oncogenic effectors, such as CDK6, MYCN, and KDM6A. Depleting linc-NeD125 in Group 4 MB cells diminishes proliferation, migration, and invasion, underscoring its tumor-promoting influence. Intriguingly, ectopic expression of linc-NeD125 in Group 3 MB cells modifies their phenotype, suggesting possible subgroup overlap in lncRNA-mediated regulation. Targeting linc-NeD125 could restore the activities of its sequestered miRNAs, reinstating their tumor-suppressive effects—an attractive strategy in high-risk and metastatic MB cases. TP73-AS1 is upregulated in Sonic Hedgehog (SHH) MBs and acts as a sponge for miR-494-3p, indirectly boosting EIF5A2 levels [21]. This mechanism reinforces proliferation and mobility. Silencing TP73-AS1 increases apoptosis and reduces proliferation *in vitro*, while *in vivo* knockdown prolongs survival in murine models. Elevated TP73-AS1 correlates with poor outcomes in TP53 wild-type SHH MB, positioning it as a potential biomarker for high-risk disease. Antisense and RNAi strategies could specifically disrupt TP73-AS1 function, impeding SHH MB progression. CRNDE is upregulated in Daoy and D341 MB cells, promoting proliferation, while its knockdown triggers S-phase arrest and apoptosis [22-24]. Although downstream targets require additional clarification, CRNDE appears to orchestrate multiple pro-growth signaling pathways, making it a candidate for broad-spectrum oncogenic inhibition. CRNDE-silenced tumors exhibit reduced mass and enhanced apoptosis, highlighting its therapeutic promise. Nonetheless, further mechanistic studies could reveal synergy with existing therapies (*e.g.*, chemotherapy). HOTAIR sponges miR-1 and miR-206, alleviating repression on YY1, a transcription factor integral to EMT [25, 26]. Enhanced proliferation, migration, and invasion are all hallmarks of HOTAIR activity in MB models. Inhibiting the HOTAIR/YY1 axis could inhibit metastatic outgrowth, an especially critical challenge in MB management. Silencing HOTAIR reduces tumor burden in animal models, validating its therapeutic tractability. CCAT1 knockdown disrupts MB cell viability and motility, which is linked to decreased activation of MAPK signaling [27]. By modulating MAPK phosphorylation states, CCAT1 contributes to oncogenic phenotypes, making it a noteworthy target for attenuating MB proliferation.

### 3.3 Summary and Future Directions for LncRNAs

Cumulatively, these lncRNAs underscore diverse routes through which MB can sustain growth and metastasis - from ECM remodeling (SPRY4-IT1) to major signaling cascades (LOXL1-AS1, CCAT1, HOTAIR) and subgroup-specific drivers (linc-NeD125, TP73-AS1). To translate these findings into clinical benefits, continued functional validation, targeted delivery of lncRNA inhibitors, and subgroup-focused preclinical trials are imperative. Enhancing our understanding of how lncRNAs interact with epigenetic factors and tumor microenvironment components could open new vistas for precision MB therapy.

### 3.4 CircRNAs in Medulloblastoma

#### 3.4.1 Major CircRNAs and their Regulatory Mechanisms

Liu *et al.* reported that CircSKA3 is upregulated in MB tissues, where it sequesters miR-520 h, protecting CDK6 from miRNA-mediated repression [28, 29]. Knockdown experiments in Daoy cells show diminished proliferation, migration, and invasion. Conversely, circSKA3 overexpression accelerates tumor progression. Silencing circSKA3 could block crucial cell-cycle regulators like CDK6, offering a potential therapeutic approach to contain MB growth and dissemination. A broader study compared MB tumor samples with normal cerebellum, identifying 33 differentially expressed circRNAs [30]. CircRNAs, such as circSKA3 and circDTL, were shown to enhance proliferation and invasion when overexpressed, and knockdown reversed these phenotypes. The identification of these dysregulated circRNAs *via* large-scale screening highlights them as markers for diagnosis, prognosis, or therapy selection. Bioinformatics analysis suggests that circRNAs intersect with pathways like TGF-β and NF-κB, linking them to tumor microenvironment modulation. Through integrative transcriptomic, metabolomic, and lipidomic profiling of CSF from MB patients, circ_463 emerged as a significantly upregulated circRNA [31]. TGF-β and TNF-α *via* NF-κB were implicated, and metabolic data revealed reprogramming in tricarboxylic acid cycle intermediates, aligning with hypoxic tumor conditions. Due to the stability of the circRNAs, measuring circ_463 levels in CSF could streamline MB detection and facilitate monitoring of disease progression or treatment response. In a study, while not distinguishing specific MB subgroups, CSF circRNA profiles robustly separated MB patients from controls—an essential step for clinical utility. circ_63706 is predominantly overexpressed in SHH MB [32]. RNA-FISH analysis of clinical samples confirmed its subgroup-specific expression. *In vitro* depletion of circ_63706 diminishes proliferation, migration, and invasion. *In vivo*, its knockdown extends survival in orthotopic MB models. Silencing circ_63706 increases ceramide and oxidized lipid levels while reducing triglycerides, suggesting that circ_63706 modulates tumor cell bioenergetics and membrane biosynthesis. Targeting circ_63706 could disrupt both proliferation and metabolic adaptation in SHH MB, a challenging subgroup often linked to specific developmental pathways. Yin *et al.* demonstrated the upregulation of circRNA_103128 in MB tissues, where it acts as a sponge for miR-129-5p, leading to increased SOX4 expression [33]. Whereas, silencing circRNA_103128 inhibits proliferation and invasion *in vitro* and slows tumor growth in murine models. Pharmacological or gene-editing approaches that curtail circRNA_103128 might restore the tumor-suppressive role of miR-129-5p, curbing MB progression.

#### 3.4.2 Future Directions for CircRNAs Research

CircRNA sequencing should be extended across WNT, SHH, Group 3, and Group 4 MBs to identify subgroup-tailored strategies. *In vivo* MB models (*e.g.*, orthotopic xenografts) can be used to confirm circRNA-mediated oncogenic or tumor-suppressive functions. CircRNA-specific interventions, such as antisense oligonucleotides, CRISPR/Cas9-based deletions, and synthetic RNA 'sponges,' should be refined. Standardized protocols for circRNA detection in CSF need to be developed to enable early diagnosis, risk stratification, and real-time monitoring of therapeutic efficacy (Table **2**).

## 4 DISCUSSION

**Fig. (2):**
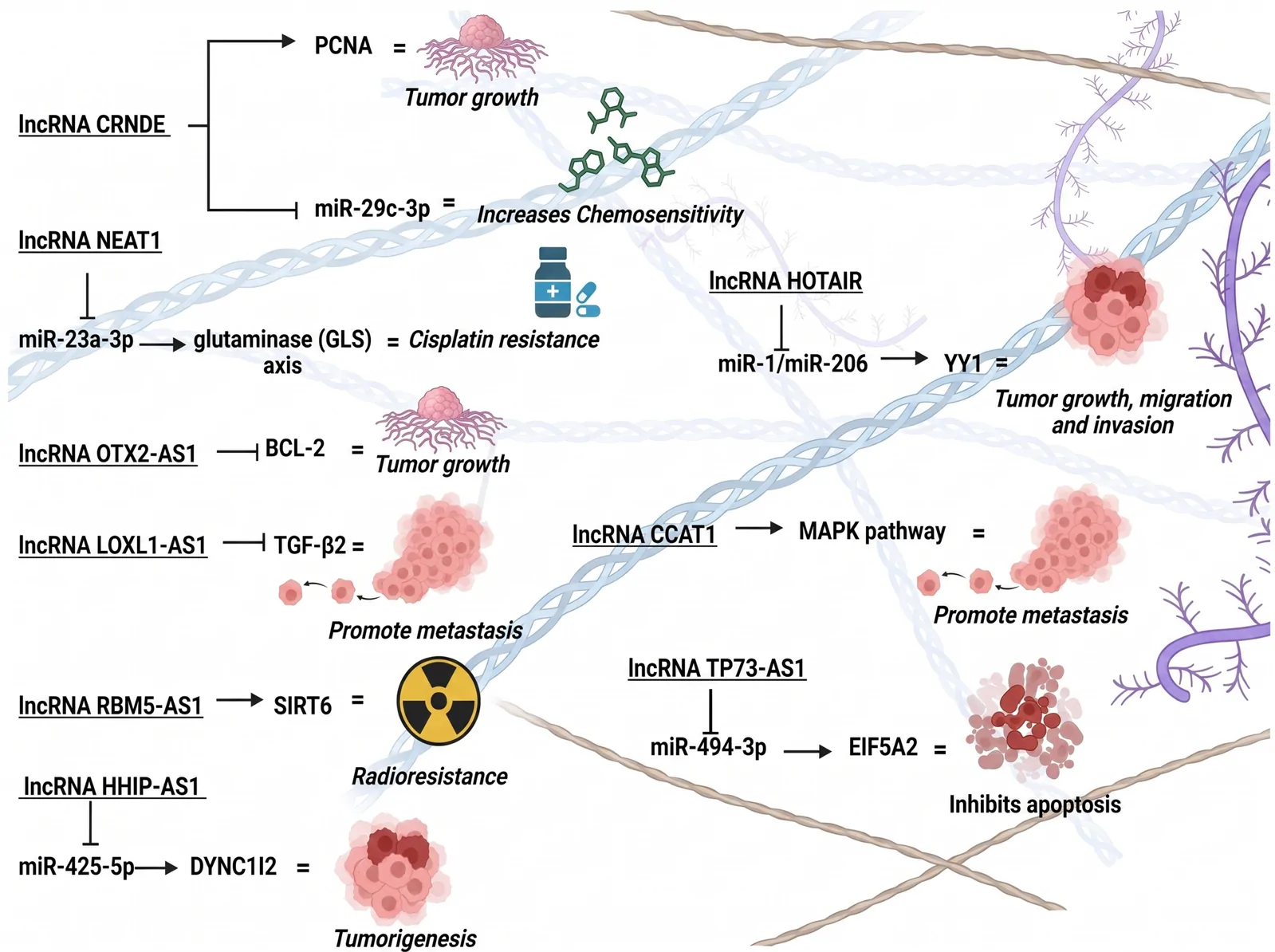
Most significantly deregulated long non-coding RNAs (lncRNAs) in medulloblastoma (MB). Several mechanisms were examined in detail, including the MAPK pathway, treatment resistance, and metastatic pathways, in order to provide a comprehensive knowledge of the involvement of lncRNAs in the pathophysiology of MB.

**Fig. (3):**
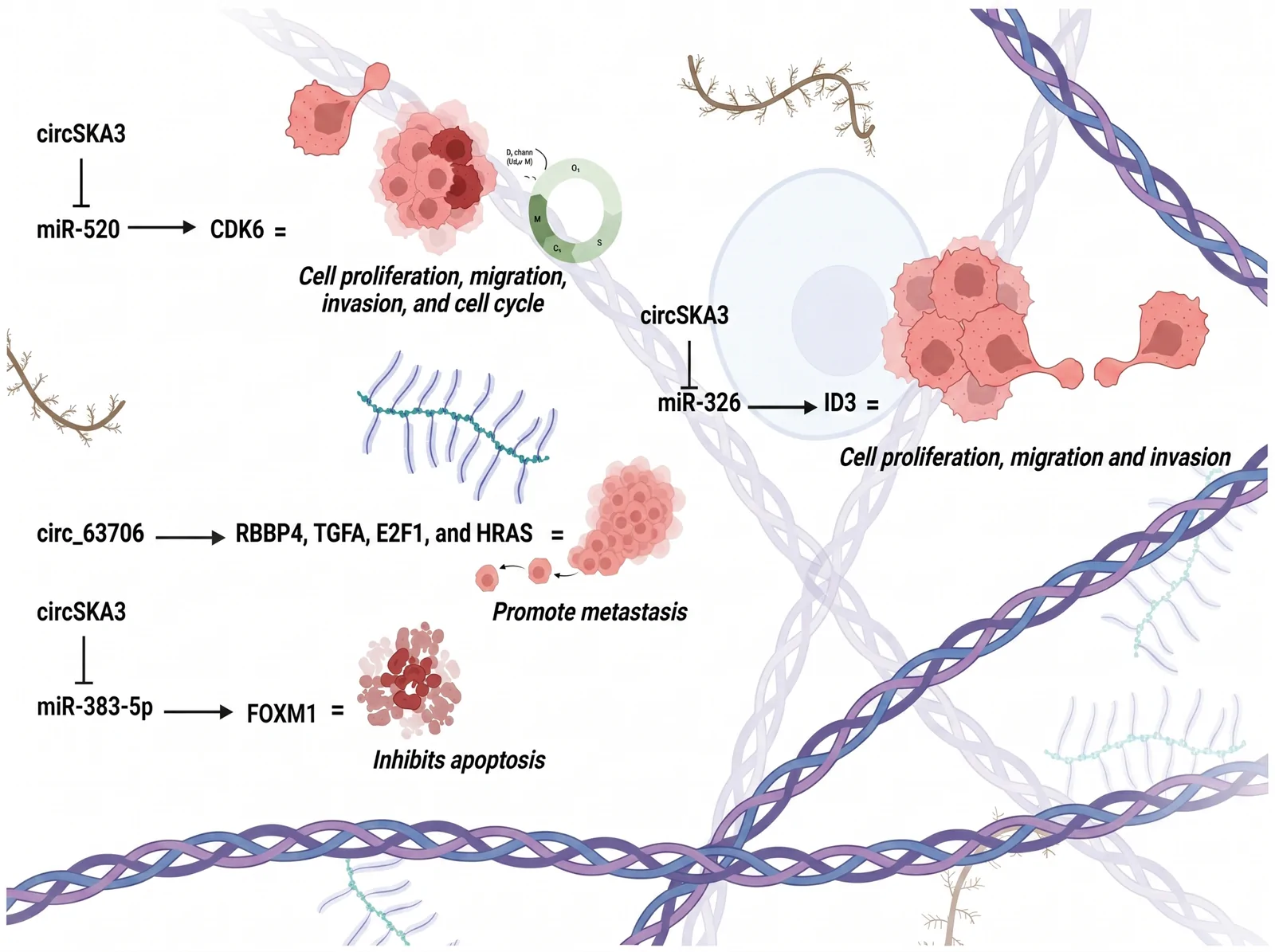
Most significantly deregulated circular RNAs (circRNAs) in medulloblastoma (MB). Several mechanisms were examined in detail, including the MAPK pathway, treatment resistance, and metastatic pathways, in order to provide a comprehensive knowledge of the involvement of circRNAs in the pathophysiology of MB.

Pediatric MBs present a formidable clinical challenge, not only due to their aggressive behavior but also because of their profound molecular heterogeneity. Recent advances highlight that ncRNAs - particularly lncRNAs and circRNAs - are central to this heterogeneity. By regulating gene expression, cell cycle processes, apoptotic pathways, Epithelial-to-Mesenchymal Transition (EMT), and even metabolic reprogramming, these ncRNAs exert a profound effect on MB progression (Figs. **2** and **3**).

## 5 DIAGNOSTIC AND PROGNOSTIC RELEVANCE

A key finding in ncRNA research is the subgroup-specific expression of certain ncRNAs. This specificity has diagnostic implications. For instance, circ_63706 is uniquely elevated in the SHH subgroup [32], potentially offering a more accurate means of subgroup classification than traditional histopathological criteria alone. Such specificity could streamline molecular risk stratification and guide therapeutic choices, enabling clinicians to identify and target the most high-risk tumors more effectively. One of the most promising aspects of circRNAs is their intrinsic stability in biological fluids, such as CSF. Since circRNAs are resistant to exonuclease degradation, they remain detectable in the bloodstream and CSF, facilitating non-invasive or minimally invasive diagnostic tests [31].

This feature opens the door to serial sampling for real- time disease monitoring, for instance, to detect early signs of recurrence or to gauge response to therapy. However, assay standardization remains a pressing requirement, and quantitative PCR methods (including RT-qPCR and digital droplet PCR) must be refined to reduce variability and enhance reproducibility across different laboratories and clinical sites. Innovative ncRNA-targeted therapies provide a way to move beyond the narrow scope of cytotoxic drugs, which often carry life-altering adverse effects. By focusing on lncRNAs and circRNAs with demonstrated oncogenic activity, novel treatments have the potential to be more selective, reducing collateral damage to healthy tissues. These short, synthetic RNA molecules bind complementary sequences on target ncRNAs, triggering degradation or preventing interactions with partner molecules. Examples include ASOs designed to inhibit oncogenic lncRNAs, such as HOTAIR or TP73-AS1. Initial preclinical data suggest that silencing these targets can impede MB progression and enhance sensitivity to existing therapies. RNA sponges are engineered to capture specific miRNAs, whereas mimics restore the function of tumor-suppressive miRNAs sequestered by oncogenic lncRNAs or circRNAs. By targeting these ncRNA-miRNA interactions, RNA sponges and mimics can disrupt tumor-promoting networks at the post-transcriptional level. CRISPR/Cas9-mediated approaches allow for highly targeted modification or complete removal of ncRNA loci. In the case of MB, this could involve excising genomic regions that encode oncogenic circRNAs or lncRNAs, thereby permanently eliminating their harmful effects. Although promising, these techniques are still under rigorous investigation to ensure precise editing and minimal off-target events. One fundamental obstacle in ncRNA research is functional redundancy within MB cells. Tumors often co-express multiple ncRNAs with overlapping functions, making it difficult to identify a single “master regulator.” Consequently, therapies targeting only one ncRNA can encounter compensatory upregulation of others, hindering long-term treatment efficacy. Safely delivering nucleic acid-based therapies across the BBB remains a major challenge. Although liposomal carriers, viral vectors, and nanoparticle-based systems show some promise, further improvements are required to achieve tumor-specific targeting, limit toxicity, and maintain stability until the payload reaches tumor cells. The intricate nature of ncRNA-protein and ncRNA-RNA interactions heightens the risk of off-target gene regulation, potentially eliciting unpredictable side effects. Therefore, robust validation processes, including *in vivo* specificity assays, are critical before any ncRNA-targeted intervention progresses to clinical trials.

## 6 FUTURE DIRECTIONS

MB subgroups (WNT, SHH, Group 3, Group 4) differ in their molecular signatures and clinical outcomes, yet the ncRNA profiles for each subgroup are far from fully understood. In-depth, subgroup-level RNA sequencing, coupled with functional assays, is vital for pinpointing high-value targets unique to each molecular subtype. Such targeted approaches could enhance the precision of MB treatments, reducing toxicity by sparing non-relevant pathways.

Combining transcriptomic, epigenomic, proteomic, and metabolomic datasets can provide a more complete picture of MB pathobiology. NcRNAs frequently interface with transcription factors, chromatin modifiers, and metabolic enzymes, underscoring the value of integrative analyses for discerning complex regulatory networks. This “pan-omics” perspective may identify synergistic vulnerabilities, guiding multi-pronged therapeutic regimens that exploit simultaneous hits to interconnected pathways. Establishing the functional importance of ncRNAs in MB requires Patient-Derived Xenografts (PDXs), Genetically Engineered Mouse Models (GEMMs), and 3D organoids. These models better approximate the tumor microenvironment and human disease progression, enabling more faithful evaluations of ncRNA-targeted therapies. Ultimately, large-scale prospective clinical trials with standardized protocols for biospecimen collection, assay normalization, and data analysis are essential. Such trials will determine whether ncRNA signatures can reliably predict patient prognosis, identify relapse earlier, and/or guide therapeutic decisions. Once validated, these ncRNA-based biomarkers could be integrated into routine clinical practice, transforming MB management through earlier detection and personalized therapy. In summary, growing evidence highlights ncRNAs as pivotal regulators in medulloblastoma, shaping both tumor biology and clinical outcomes. While challenges, such as delivery barriers and functional redundancy, remain, rigorous ongoing research holds promise for leveraging these ncRNAs into cutting-edge diagnostics and therapies that can significantly improve survival and quality of life for children affected by this aggressive malignancy.

## CONCLUSION

Pediatric MB is a molecularly diverse and clinically challenging brain malignancy, underscoring the urgent need for innovative diagnostics and therapeutic strategies. Recent studies have identified ncRNAs, including miRNAs, lncRNAs, and circRNAs, as pivotal regulators of the onset and progression of MB. These ncRNAs influence a myriad of oncogenic processes, such as cell cycle regulation, EMT, and metabolic reprogramming, through mechanisms that include miRNA sponging, modulation of transcription factors, and direct interactions with signaling pathways [34-36]. This review has synthesized recent findings that highlight the multifaceted contributions of ncRNAs, including miRNAs, lncRNAs, and circRNAs, to the pathogenesis, diagnosis, and treatment potential of medulloblastoma [37-40]. Investigations into lncRNAs like HOTAIR and TP73-AS1 reveal their capacity to modulate tumor suppressors and oncogenes by binding specific miRNAs (*e.g.*, miR-129-5p, miR-494-3p) or transcription factors (*e.g.*, SOX4, YY1, CDK6). These interactions drive tumor cell survival, proliferation, and metastasis. Due to their closed-loop structures, circRNAs (*e.g.*, circ-SKA3, circ_63706) exhibit high stability and often function as miRNA sponges, sequestering tumor-suppressive miRNAs to sustain oncogenic signaling. Subgroup-specific circRNAs underscore the heterogeneity of MB and highlight the potential for targeted subgroup-based therapies.

NcRNAs hold promise as minimally invasive biomarkers. Their stability in CSF allows real-time disease monitoring, early detection of relapse, and improved molecular classification of MB subgroups [41, 42]. Advances in RNA sequencing and multi-omics profiling have accelerated the identification of subgroup-specific ncRNA signatures, moving toward refined risk stratification. Novel interventions, including ASOs, RNA sponges/mimics, and CRISPR/Cas9 systems, offer avenues for suppressing or editing oncogenic ncRNAs. Such approaches could complement or even reduce reliance on traditional cytotoxic treatments and thereby mitigate long-term toxicities. Despite extensive correlational data, many ncRNAs require deeper mechanistic validation in robust preclinical models (*e.g.*, patient-derived xenografts and genetically engineered mouse models). Nucleic acid-based therapies face delivery hurdles, particularly crossing the blood-brain barrier and achieving tumor specificity without eliciting systemic toxicity. Clinical assays must be reproducible and cost-effective, ensuring that ncRNA profiling, including liquid biopsy approaches, can be seamlessly adopted in routine pediatric neuro-oncology practices. Medulloblastomas frequently express multiple, overlapping ncRNAs that can compensate for each other when one is inhibited, complicating the design of single-target approaches. Since MB subgroups (WNT, SHH, Group 3, Group 4) differ genetically and clinically, ncRNA profiling should be broadened to pinpoint subgroup-unique targets. Tailoring therapies to these molecular differences holds promise for enhanced efficacy. Combining ncRNA-targeted interventions with existing modalities, such as chemo- and radiotherapy, could improve outcomes while potentially reducing drug doses and long-term side effects. Rigorous, well-designed trials are essential for assessing the real-world impact of ncRNA-based diagnostics and therapies. Standardized protocols for biospecimen collection, data normalization, and outcome measures will be crucial for validating the clinical utility of ncRNA signatures [43, 44]. Developing sophisticated nanoparticle carriers, viral vectors, or exosome-based systems may circumvent current delivery obstacles and facilitate the precise targeting of intracranial tumors. NcRNAs are increasingly recognized as central orchestrators of MB pathogenesis and progression, offering unprecedented opportunities for precision medicine. As functional studies deepen our understanding of ncRNA networks, therapeutic strategies that selectively modulate these molecules will likely emerge.

By incorporating ncRNA profiling into standard diagnostic workflows and refining targeted interventions, we can aim for more personalized and less toxic treatments for children with this aggressive disease. Ultimately, harnessing the potential of ncRNAs could transform MB management, improving survival rates and quality of life for pediatric patients worldwide.

## Figures and Tables

**Table 1:** Summary of the roles of key lncRNAs involved in medulloblastoma.

**lncRNA**	**Target Pathway**	**Molecular Functions**	**Biological Roles in Medulloblastoma Cell**	**References**
**SPRY4-IT1**	Unknown	(miR-103, miR-107 and miR-548m)	Promotion of cell proliferation, migration, and invasion	[18]
**LOXL1-AS1**	PI3K/AKT pathway	(miR-19a-3p, miR-19b-3p and miR-106a-5p)	Promotion of cell proliferation and metastasis	[19]
**Linc-NeD125**	Unknown	miRNA sponge	Promotion of cell proliferation, migration, and invasion	[20]
**NKX2-2AS**	SHH pathway	Unknown	Suppression of cell proliferation, migration, and invasion	[23]
**CRNDE**	Unknown	Unknown	Promotion of cell cycle progression	[24]
**UCA1**	Unknown	Host gene for miRNA	Promotion of cell proliferation and migration	[25]
**CCAT1**	MAPK pathway	Unknown	Promotion of cell proliferation and metastasis	[27]

**Table 2:** Summary of the roles of key circRNAs involved in medulloblastoma.

**circRNA**	**Mechanism**	**Function**	**References**
**circSKA3**	Acts as a 'sponge' for miR-520 h, modulating CDK6 expression.	Regulates CDK6 expression by sequestering miR-520 h, promoting cell proliferation, migration, invasion, and cell cycle progression.	[29]
**circ-SKA3** **circ-DTL**	Regulates host gene expression, affecting proliferation, migration, and invasion.	Regulate tumor cell proliferation and invasiveness, and serve as biomarkers for MB diagnosis and therapeutic targets.	[30]
**circ_463**	Differentiates MB from non-cancerous conditions *via* CSF analysis.	Identified in CSF as a potential diagnostic biomarker linked to metabolic alterations due to MB.	[31]
**circ_63706**	Impacts lipid metabolism, promoting tumor progression.	Promotes tumor growth and alters lipid metabolism, specific to the SHH subgroup of MB.	[32]
**circRNA_103128**	Sponges miR-129-5p, facilitating SOX4 upregulation.	Acts as a molecular sponge for miR-129-5p, facilitating SOX4 upregulation and promoting tumorigenesis and progression.	[33]

## LIST OF ABBREVIATIONS

ECM: Extracellular Matrix
CSF: Cerebrospinal Fluid
SHH: Sonic Hedgehog
ncRNAs: Non-coding RNAs
lncRNAs: Long Non-coding RNAs
circRNAs: Circular RNAs
MBs: Medulloblastomas
